# Antipsychotic Use and Psychiatric Hospitalization in First-Episode Non-affective Psychosis and Cannabis Use Disorder: A Swedish Nationwide Cohort Study

**DOI:** 10.1093/schbul/sbae034

**Published:** 2024-03-26

**Authors:** Alexander Denissoff, Heidi Taipale, Jari Tiihonen, Marta Di Forti, Ellenor Mittendorfer-Rutz, Antti Tanskanen, Antti Mustonen, Solja Niemelä

**Affiliations:** Department of Psychiatry, Faculty of Medicine, University of Turku, Turku, Finland; Addiction Psychiatry Unit, Department of Psychiatry, Turku University Hospital, The Wellbeing Services County of Southwest Finland, Turku, Finland; Department of Forensic Psychiatry, University of Eastern Finland, Niuvanniemi Hospital, Kuopio, Finland; Department of Clinical Neuroscience, Karolinska Institutet, Stockholm, Sweden; School of Pharmacy, University of Eastern Finland, Kuopio, Finland; Department of Forensic Psychiatry, University of Eastern Finland, Niuvanniemi Hospital, Kuopio, Finland; Department of Clinical Neuroscience, Karolinska Institutet, Stockholm, Sweden; Department of Social Genetics and Developmental Psychiatry, IoPPN, King’s College London, London, England; Department of Clinical Neuroscience, Karolinska Institutet, Stockholm, Sweden; Department of Forensic Psychiatry, University of Eastern Finland, Niuvanniemi Hospital, Kuopio, Finland; Department of Clinical Neuroscience, Karolinska Institutet, Stockholm, Sweden; Faculty of Medicine and Health Technology, Tampere University, Tampere, Finland; Department of Psychiatry, Seinäjoki Central Hospital, Seinäjoki, Finland; Department of Psychiatry, Faculty of Medicine, University of Turku, Turku, Finland; Addiction Psychiatry Unit, Department of Psychiatry, Turku University Hospital, The Wellbeing Services County of Southwest Finland, Turku, Finland

**Keywords:** cannabis, psychosis, first-episode, antipsychotic

## Abstract

**Background and Hypothesis:**

There is a paucity of research on treatment outcomes of patients with psychosis and cannabis use disorder (CUD). We aimed to compare the effectiveness of antipsychotics in reducing the risk of hospitalization in patients with first-episode psychosis (FEP) and co-occurring CUD.

**Study Design:**

We utilized a nationwide Swedish cohort of patients with longitudinal register data from the year 2006 to 2021. Participants were patients with FEP and co-occurring CUD (*n* = 1820, 84.73% men, mean age 26.80 years, SD 8.25 years). The main outcome was hospitalization due to psychotic relapse. Hospitalization due to any psychiatric disorder or substance use disorder (SUD) were examined as secondary outcomes. Within-individual Cox regression models were used to study these associations.

**Study Results:**

Use of any antipsychotic was associated with a 33% risk reduction of psychotic relapse (aHR = 0.67; 95% CI 0.60–0.75). Clozapine (0.43; 0.29–0.64), long-acting injectable (LAI) formulations of risperidone (0.40; 0.22–0.71), aripiprazole (0.42; 0.27–0.65), and paliperidone (0.46; 0.30–0.69) were associated with the lowest risk of relapse. The association between the LAI formulation of olanzapine and hospitalization due to psychosis was statistically non-significant (0.61; 0.35–1.05). Clozapine was associated with an 86% risk reduction of hospitalization due to SUD (0.14; 0.05–0.44). Of oral non-clozapine antipsychotics, aripiprazole was associated with the lowest risk of hospitalization due to psychotic relapse (0.61; 0.45–0.83).

**Conclusions:**

These findings support the use of clozapine, LAI formulations of second-generation antipsychotics other than olanzapine, or oral aripiprazole to prevent hospitalization in FEP and co-occurring CUD.

## Introduction

Cannabis use is ubiquitous among first-episode psychosis (FEP) patients with a significant proportion continuing use after recovery.^1^ Comorbid cannabis use disorder (CUD) in this patient group is common, as prevalence figures of current CUD of 10%–50% have been reported in clinical and register-based FEP cohorts.^2–4^ Continued cannabis use after FEP has been associated with elevated severity of psychotic symptoms^5–9^ and increased frequency and duration of relapses.^10–13^ Current or former cannabis use at FEP has been found to be associated with a greater burden of lifetime-inpatient care,^14–16^ and subsequent treatment-resistant psychosis.^17^ However, cannabis use is also associated with non-adherence to antipsychotics in FEP.^10,16^ Thus, optimizing pharmacotherapy in this dual disorder condition is crucial.

Current knowledge on the efficacy of antipsychotic treatment among persons with FEP and comorbid CUD is scarce. Real-world treatment outcomes of patients with psychotic disorders and substance use disorder (SUD) have been examined in nationwide register-based studies.^18^ However, we are not aware of such studies focusing exclusively on patients with FEP and co-occurring CUD. The few existing clinical trials examining the efficacy of antipsychotic drugs in dual disorders have merged groups of patients with psychosis and co-occurring cannabis use or CUD and groups of psychosis patients inflicted by other types of substance use comorbidities.^19–21^ So far, only three small clinical trials have focused exclusively on psychotic disorders with co-occurring CUD.^22–24^ None of these studies focused on severe outcomes such as hospitalization due to a psychotic relapse, and durations of follow-up ranged only from 12 weeks to 12 months. A limited number of antipsychotic drugs were encompassed in this body of literature and only one of these studies focused on FEP specifically.^23^ Yet, improving outcomes of FEP patients with dual disorders is of paramount importance, as relapse after the index psychotic episode is associated with adverse clinical outcomes.^25^

In this study, we aim to examine the real-world treatment outcomes of patients with FEP and co-occurring CUD (*n* = 1820) utilizing register-linkage data from a Swedish nationwide cohort from year 2006 to 2021. The data utilized contains information on all antipsychotic agents used in clinical practice enhancing the generalizability of the results. To overcome selection bias, a within-individual design is utilized in the main analyses.^26^ We focus on the comparative effectiveness of antipsychotics with respect to clinically relevant outcomes such as hospitalization due to psychotic relapse, hospitalization due to any psychiatric cause, or hospitalization due to SUD, with a follow-up up to 15 years.

## Methods

### Study Population

Prospective data was acquired from Swedish nationwide registers including the National Patient Register, the Longitudinal Integration Database for Health Insurance (LISA), Labor Market Studies register, and the Micro Data for Analyses of Social Insurance (MiDAS) register. The study population consisted of all persons aged 16–64 years residing in Sweden with a registered first treatment contact due to non-affective psychotic disorder (used as a proxy for FEP) (ICD-10 codes F2x.1-9) and co-occurring CUD defined as any cannabis use -related diagnosis (ICD-10 codes F12.0-F12.9) between July 2006 to December 2021 in any of the above-mentioned registers providing data on specialized health care, sickness absences, and disability pensions. Data regarding sickness absences were derived from the MiDAS register, which is managed by the Swedish Social Insurance Agency and provides information on periods during which individuals have received sickness benefits due to health-related incapacity for work.^27^ In Sweden, each permanent resident is assigned a unique social security number by which information can be linked between different registries. For CUD to be classified as co-occurring, the time of registration of this diagnosis had to be at most two weeks prior or no more than 2 weeks later than the registration of the respective FEP diagnosis. Persons with previous diagnosis of non-affective psychotic disorder since 1969 were excluded from analyses.

We utilized data from the REWHARD consortium supported by the Swedish Research Council (grant number 2021-00154). The research project was approved by the Regional Ethics Board of Stockholm, Karolinska Institutet, Stockholm, Sweden (decision 2007/762-31 and Dnr 2021-06441-02).

### Exposure

Data on dispensed antipsychotics were acquired from the Prescribed Drug Register (PDR) from July 2005 to December 2021. Information on medication use is categorized in the PDR according to the Anatomic Therapeutic Chemical (ATC) classification. Monotherapies of different antipsychotic agents (ATC codes N05A excluding lithium N05AN01) were analyzed by drug formulation, in addition to AP polytherapy (concomitant use of two or more antipsychotics). The reference condition or group in each within-person or between-person analysis was the non-use of any antipsychotic. Drug use periods were estimated utilizing the PRE2DUP approach described elsewhere.^28^ All exposures were defined time-dependently, that is, changes in use vs non-use were followed up and updated in the models. Data on other psychotropics were gathered from the PDR, modeled with PRE2DUP, and included in the analyses as time-varying exposures. These included antidepressants (N06A), mood stabilizers (N03AF01, N03AG01, N03AX09, N05AN01), benzodiazepines and related pharmaceuticals (N05BA, N05CD, N05CF), and ADHD medications (N06BA), and they were coded as use vs non-use of each medication class. Medications with fewer than 20 outcomes were not reported.

### Outcomes

The main outcomes of this study were (1) hospitalization due to psychotic disorder (ICD-10 diagnoses F2x.xx, relapse), (2) hospitalization due to any psychiatric disorder (F00-F99), and (3) hospitalization due to any SUD (ICD-10 F10–F19). ICD-10 codes denoting these hospitalizations (inpatient stay at least overnight) were obtained from the National Patient Register, a database with nationwide coverage on all specialized inpatient care.^29^ Information on deaths (*n* = 3) and times of emigration from Sweden (*n* = 115), which were used as censoring points in the analyses, were obtained from the LISA register and the Registry for Causes of Death.

### Statistical Methods

We applied Cox regression analysis with hazard ratios (HR) and 95% confidence intervals (CI) to study the association of the use of different antipsychotics with the outcomes of interest. The reference category was the non-use of any antipsychotic agents. A within-individual approach was taken to eliminate selection bias.^30^ In this design, each person acts as his/her own control, and all time-invariant factors are eliminated in the design. The follow-up time is reset to zero after each outcome event. The stratified Cox models were adjusted for time-varying factors which were the use of other psychotropic medications, time since cohort entry, and temporal order of antipsychotic treatments.

Traditional multivariable between-person Cox-regression analyses were also conducted to examine the association between the use of specific second-generation antipsychotics (SGAs), (ie, olanzapine, quetiapine, risperidone, and aripiprazole), antipsychotic polytherapy, any LAI and other antipsychotics (excluding the aforementioned SGA:s) with hospitalization due to psychotic relapse.

Statistical analyses were performed using SAS version 9.4. Forest plot figures were created using R version 4.1.01.

## Results

Demographic characteristics of the sample are presented in table 1. The sample totaled 1820 individuals (84.7%, *n* = 1542 male) with an average age at baseline of 26.8 years (SD 8.3 years). At study entry, 21.3% of the participants had been on sickness absence during the previous year and 9.7% were current disability pension recipients. The prevalence distribution of co-occurring CUD diagnoses at the time of FEP was 33.9% for F12.1 Harmful cannabis use, 32.4% for F12.5 Cannabis induced psychosis (CIP), 20.6% for F12.2 Cannabis dependence and 13.1% for other cannabis use-related diagnoses, respectively. During the follow-up, 63.6% (1158/1820) of all the participants were re-diagnosed with any cannabis use-related diagnosis (F12.x), and 46.9% of all the participants (854/1820) specifically with F12.1 Harmful cannabis use or F12.2 Cannabis dependence.

**Table 1.** Demographic Characteristics of Cohort

	Frequency	Percent
Calendar year of diagnosis		
2006–2011	407	22.36
2012–2017	838	46.04
2018–2021	575	31.59
Age		
16–19	206	11.32
20–24	710	39.01
25–29	446	24.51
≥30	458	25.16
Sex		
Female	278	15.27
Male	1542	84.73
Born in Sweden		
No	555	30.49
Yes	1265	69.51
Education		
Unknown	115	6.32
Low	725	39.84
Medium	793	43.57
High	187	10.27
Income from work		
No	809	44.45
Yes	1011	55.55
Sickness absence previous year		
No	1433	78.74
1–90 days	284	15.6
≥90 days	103	5.66
Disability pension at cohort entry		
No	1643	90.27
Yes	177	9.73

### Risk of Hospitalization Due to Relapse to Psychosis

**Fig. 1.**
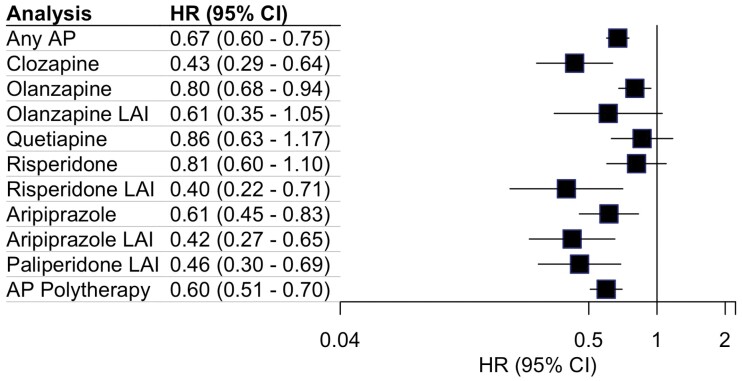
Association of second-generation antipsychotics with subsequent hospitalization due to psychotic relapse.

In the cohort, 1111 of 1820 persons (61%) were hospitalized due to psychotic relapse (ICD-10 diagnosis of F20–F29). The mean follow-up time in this analysis was 6.13 years (SD 4.12 years). The number of users, number of events, and mean follow-up times are presented in Supplementary table 1. Oral olanzapine was the most frequently prescribed antipsychotic (*n* = 1038). The results of the within-individual analysis models for SGAs and antipsychotic polytherapy regarding this outcome are presented in figure 1. Compared with non-use, the use of any antipsychotic was associated with a 33% risk reduction of psychotic relapse (aHR = 0.67; 95% CI 0.60–0.75). Long-acting injectable (LAI) formulations of risperidone (0.40; 0.22–0.71), aripiprazole (0.42; 0.27–0.65), and paliperidone (0.46; 0.30–0.69), and oral clozapine (0.43; 0.29–0.64) were associated with the lowest risk of relapse. Effect sizes for second-generation (SGA) LAI formulations were uniformly greater than for their respective oral formulations. In contrast to other LAI, the LAI formulation of olanzapine did not reach statistical significance for effectiveness in preventing hospitalization due to psychosis (0.61; 0.35–1.05). Of oral non-clozapine antipsychotics, aripiprazole was found to be associated with the lowest risk of relapse (0.61; 0.45–0.83). The effect size of the oral formulation of olanzapine was moderate (0.80; 0.68–0.94). Quetiapine was not found to be efficacious in preventing psychotic relapse (0.86; 0.63–1.17). Antipsychotic (AP) polytherapy was associated with a 40% risk reduction of psychotic relapse (0.60; 0.51–0.70).

The results of the between-person analyses are presented in Supplementary table 2. The HRs for first-generation antipsychotics with at least 20 events are presented in Supplementary table 3 for all outcomes.

### Risk of Hospitalization Due to Any Psychiatric Diagnosis

**Fig. 2.**
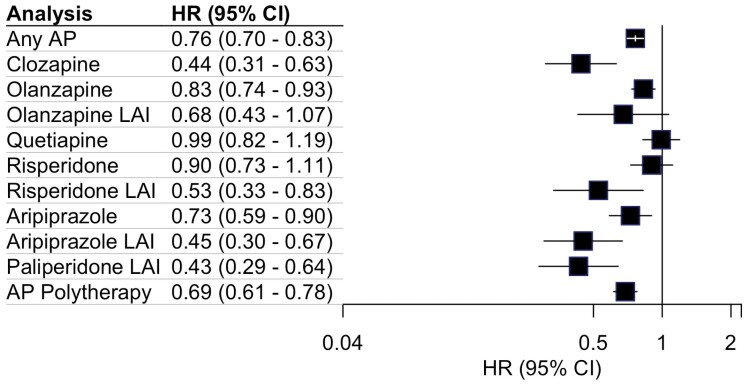
Association of second-generation antipsychotics with subsequent hospitalization due to any psychiatric disorder.

In the cohort, 1376 of 1820 persons (76%) were hospitalized due to any psychiatric reason at least once during follow-up. The number of users, number of events, and mean follow-up times are presented in Supplementary table 4. Results of the within-individual models for SGAs and AP polytherapy regarding this outcome are presented in figure 2. Compared with non-use, the use of any antipsychotic drug was associated with a 24% decreased risk of hospitalization due to any psychiatric diagnosis (0.76; 0.70–0.83). The lowest risk was found for LAI formulations of aripiprazole (0.45; 0.30–0.67) and paliperidone (0.43; 0.29–0.64), and similar to oral clozapine (0.44; 0.31–0.60). The LAI formulation of olanzapine did not reach statistical significance for effectiveness in preventing hospitalization due to any psychiatric diagnosis (0.68; 0.43–1.07). Of all non-clozapine antipsychotics, oral aripiprazole was associated with the lowest risk of hospitalization due to any psychiatric diagnosis (0.73; 0.59–0.90). The aHR of the oral formulation of olanzapine was moderate (0.83; 0.74–0.93). Oral formulations of quetiapine (0.99; 0.82–1.19) and risperidone (0.90; 0.73–1.11) failed to achieve statistical significance with respect to this outcome. AP polytherapy was associated with a 31% risk reduction of subsequent hospitalization due to any psychiatric disorder (0.69; 0.61–0.78).

### Risk of Hospitalization Due to SUD-related Cause

**Fig. 3.**
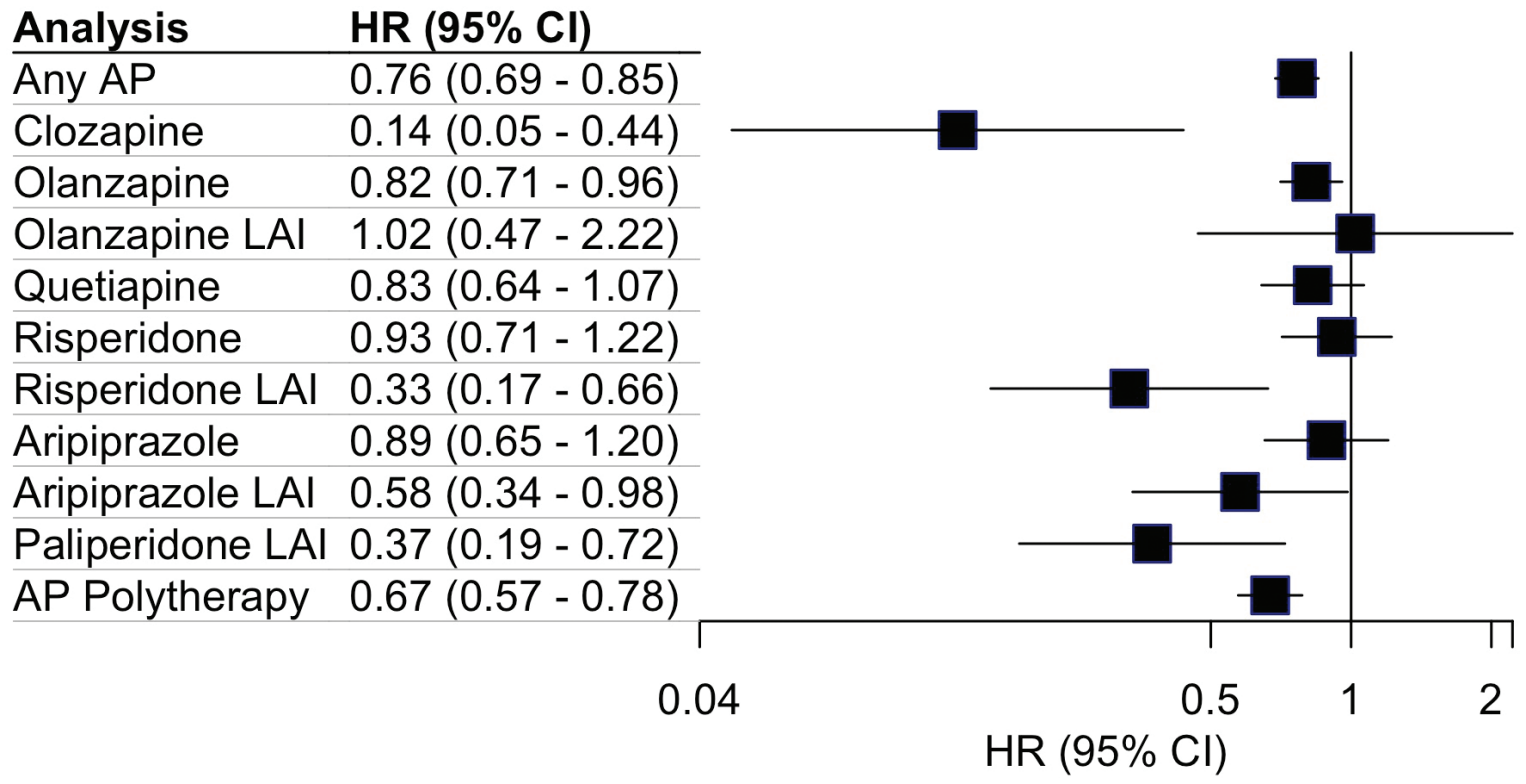
Association of second-generation antipsychotics with subsequent hospitalization due to any substance use disorder.

In the cohort, 1143 of 1820 persons (63%) were hospitalized due to substance use disorder (SUD) at least once by the end of follow-up. The number of users, number of events and mean follow-up times are presented in Supplementary table 5. The distribution of different SUD types for this outcome is provided in the Supplementary table 6. The results of the within-individual model for this outcome are presented in figure 3. Compared with non-use, use of any antipsychotic was associated with a 24% risk reduction of hospitalization due to SUD (0.76; 0.69–0.85). Clozapine was associated with a substantial 86% decreased risk of subsequent hospitalization due to SUD (0.14; 0.05–0.44), the LAI formulations of paliperidone (0.37; 0.19–0.72), and risperidone (0.33; 0.17–0.66) followed clozapine in terms of comparative effectiveness with respect to this outcome. AP polytherapy was associated with a 33% risk reduction of subsequent hospitalization due to any SUD (0.67; 0.57–0.78).

## Discussion

This is the first nationally representative register-linkage study focusing on treatment outcomes in first-episode psychosis (FEP) with co-occurring cannabis use disorder (CUD). We found that the use of any antipsychotic was associated with a 33% decreased risk of relapse to psychosis compared to the non-use of antipsychotics. Most importantly, we found that LAI formulations of second-generation antipsychotics (SGAs) other than olanzapine were associated with the lowest risk of psychotic relapse and risk of any psychiatric hospitalization, in addition to oral clozapine. Clozapine seemed to be particularly effective in reducing the risk of subsequent SUD hospitalizations. Lastly, oral aripiprazole seemed to be the most effective oral non-clozapine SGA in reducing the risk of psychotic relapses and psychiatric hospitalizations.

Only three previously published studies have focused exclusively on antipsychotic efficacy in psychotic disorders with co-occurring CUD.^22–24^ None of these studies focused on severe outcomes such as hospitalization due to relapse to psychosis. Only one previous study focused on FEP patients and found risperidone to be non-inferior to olanzapine in terms of effectiveness in this patient group.^23^ That study had a reduction of psychopathology as its main outcome, whereas the other two studies focused primarily on the change in cannabis consumption.^22,24^

We found LAI formulations of SGAs excluding olanzapine to be equally efficacious as clozapine in reducing the risk of psychotic relapse and any psychiatric hospitalization in FEP patients. A Swedish nationwide register-linkage study focusing on treatment outcomes in persons with schizophrenia with lifetime SUD has been published previously.^18^ There, the effectiveness of LAI antipsychotics in reducing the risk of subsequent psychiatric hospitalization was found to be more moderate than in our study. Also, clozapine was clearly associated with the lowest risk of subsequent psychiatric hospitalization in that study. However, that study cohort comprised of specifically schizophrenia patients, and many of them already had a long duration of illness. This is of significance, as treatment response in antipsychotic therapy is less likely after relapse to psychosis.^31^ Moreover, a recent RCT found LAIs to be superior to oral formulations of antipsychotics other than clozapine in early-phase schizophrenia,^32^ and similar findings have been reported in a Finnish register-linkage study.^33^ As cannabis use is associated with non-adherence in FEP,^10^ SGA LAIs should be favored in patients with FEP and co-occurring cannabis use.

We found clozapine to be associated with the lowest risk of hospitalization due to SUD. Findings from recent meta-analyses indicate clozapine to be superior to other antipsychotics in reducing substance use^19^ and promoting abstinence^21^ in patients with psychotic disorders and comorbid SUDs. Clozapine was also found to be superior to risperidone in reducing cue reactivity in a randomized fMRI study focusing on patients with schizophrenia and comorbid CUD.^34^ Most importantly, in the Swedish register-linkage study described previously, clozapine was associated with a many folds more modest decrease in risk of subsequent hospitalization due to SUD (a 29% decrease in risk) than in our study.^18^ Our finding thus highlights the special utility of early use of clozapine in improving the outcomes of specifically FEP patients with CUD. Our finding is also in line with the results of a previously published RCT focusing specifically on change in cannabis use.^22^ There, patients with schizophrenia and CUD were randomized to stay on their current antipsychotic or switch to clozapine. Switching to clozapine was found to be associated with a reduction of cannabis consumption. However, in another RCT focusing on this outcome, clozapine was not superior to ziprasidone in reducing cannabis use.^24^ It has been proposed that that findings from non-randomized studies pointing to the superior effectiveness of clozapine in improving outcomes of co-occurring SUDs may be influenced by various confounding factors^35^: It might be that clinicians prescribe clozapine mainly to patients who present with better adherence to treatment (and mandatory monthly monitoring due to neutropenia risk) and less severe substance use. Patients willing to initiate clozapine treatment may also be more ready to engage in the treatment of their comorbid SUD. Thus, it is possible that the results of clozapine represent a somewhat clinically selected patient group.

In line with previous findings,^36^ antipsychotic polytherapy was found to be associated with a reduced risk of all outcomes studied. This is of significance, as previous findings on the efficacy of antipsychotic polytherapy have been mixed^37,38^and this form of treatment has been discouraged in clinical guidelines.^39^ It has been suggested that the observed effectiveness of antipsychotic polypharmacy might be related to the increased likelihood of using at least one antipsychotic agent.^36^ However, monotherapy with SGA LAIs other than olanzapine and oral clozapine were associated with a lower risk of any subsequent outcome than antipsychotic polytherapy. Lastly, aripiprazole was found to be the most effective oral non-clozapine antipsychotic. It has been proposed that patients with dual disorders might benefit from the modulation of dopaminergic pathways induced by partial agonists.^40^ In accordance with this, findings from RCTs indicate oral or LAI formulations of aripiprazole to be superior to oral perphenazine or the LAI formulation of paliperidone in reducing cravings.^41,42^ It is also plausible that favorable outcomes of aripiprazole might be due to the exceptional tolerability of this agent compared to other antipsychotics.^43^

Oral olanzapine was the most frequently prescribed antipsychotic in our study cohort. However, it was found to be associated with only a modest risk reduction with respect to any outcome studied. This was surprising, as meta-analytic evidence points to olanzapine and amisulpride to be more efficacious than other non-clozapine agents in FEP.^44^ While power issues likely explain the statistically non-significant associations of the LAI form of olanzapine with the outcomes studied, the reason for the modest efficacy of oral olanzapine is less clear. Weight gain is particularly pronounced in young patients using olanzapine,^45^ and this adverse effect has been associated with non-adherence to antipsychotic treatment.^46^

This study presents with several strengths: Focusing exclusively on co-occurring CUD among persons with FEP is to be regarded as a strength, as cannabis use, in particular, has been associated with more deleterious outcomes than the use of other substances within this population^10^ Nationwide register-based data with information on all patients presenting with this type of dual diagnosis and all antipsychotics used provides with exceptional generalizability of these results. The use of within-person analyses reduces the possibility of selection bias. Attrition was small as very few patients emigrated or died during follow-up.

However, there are limitations: Not having information on cannabis use trajectories during follow-up is to be regarded as a limitation, as continued cannabis use has been associated with poorer outcomes than discontinued use in FEP.^5–9^ However, 63.6% of the participants were re-diagnosed with CUD (F12.x) by the end of the follow-up. Thus, it is reasonable to believe that a considerable proportion of patients continued their cannabis use after their first diagnosis of psychotic disorder. As underdiagnosis of SUD is common,^47^ it is possible that some patients with CUD may have not been included in the study cohort for this reason. As we focused on FEP patients with a diagnosis of co-occurring CUD made in clinical practice, our findings are not necessarily representative of patients with less severe cannabis use. Finally, CUD was defined to encompass all F12.x diagnoses in this study rather than F12.1-2 only. Patients diagnosed concomitantly with non-affective psychosis (F2x) and CIP (F12.5) were included in the analyses, which presents as a diagnostic conundrum. However, a diagnosis of CIP evidently implies the fulfillment of the criteria of F12.1 Harmful cannabis use.

## Conclusions

In FEP patients with co-occurring CUD, LAI formulations of SGAs other than olanzapine were associated with a similarly decreased risk as clozapine in terms of hospitalization due to psychotic relapse or other psychiatric disorders. While clozapine was found to be associated with a substantial risk reduction of subsequent hospitalization due to SUDs, this finding might not be void of selection bias. Thus, these findings encourage the early use of SGA LAIs as an important secondary prevention strategy to reduce rates of hospitalization in FEP patients with comorbid CUD.

## Supplementary Material

Supplementary material is available at https://academic.oup.com/schizophreniabulletin/.

sbae034_suppl_Supplementary_Tables
