# Supplementary material for: Antipsychotic Use and Psychiatric Hospitalization in First-Episode Non-affective Psychosis and Cannabis Use Disorder: A Swedish Nationwide Cohort Study
Source: Schizophr Bull. 2024 Mar 26;50(6):1287–94. doi: 10.1093/schbul/sbae034 (PMC11578669; doi:10.1093/schbul/sbae034)
Supplement: sbae034_suppl_Supplementary_Tables [file sbae034_suppl_supplementary_tables.docx]

**Online supplement table 1. Number of users, total person years and total number of events for hospitalization due to psychosis outcome**

**Online supplement table 2. aHR:s and confidence intervals for between-person analyses on use of antipsychotics and risk of hospitalization due to psychosis relapse**

**Online supplement table 3. aHR:s and confidence intervals for FGA:s**

**Online supplement table 4. Number of users, total person years and total number of events for hospitalization due to any psychiatric disorder**

**Online supplement table 5. Number of users, total person years and total number of events for hospitalization due to substance use disorder outcome**

**Online supplement table 6. Distribution of Substance use disorder hospitalizations at follow-up**

| **Antipsychotic** | **Number of users** | | **Total person years** | | **Total number of events** | |
| --- | --- | --- | --- | --- | --- | --- |
| Clozapine | 73 | 146.49 | | 53 | |  |
| Olanzapine | 1038 | 891.39 | | 413 | |  |
| Olanzapine LAI | 63 | 51.18 | | 24 | |  |
| Quetiapine | 361 | 312.13 | | 104 | |  |
| Risperidone | 286 | 190.53 | | 98 | |  |
| Risperidone LAI | 73 | 66.93 | | 23 | |  |
| Aripiprazole | 412 | 328.3 | | 118 | |  |
| Aripiprazole LAI | 101 | 112.99 | | 38 | |  |
| Paliperidone LAI | 115 | 108.15 | | 41 | |  |
| Ap polytherapy | 818 | 851.46 | | 580 | |  |
| Levomepromazine | 135 | 59.93 | | 31 | |  |
| Perphenazine LAI | 50 | 40.58 | | 32 | |  |
| Haloperidol | 188 | 118.42 | | 74 | |  |
| Haloperidol LAI | 49 | 48.63 | | 35 | |  |
| Zuclopenthixol LAI | 70 | 56.92 | | 37 | |  |

Online supplement table 1.Number of users, total person years and total number of events for hospitalization due to psychosis outcome

|  |  |  |  |
| --- | --- | --- | --- |

Online supplement table 2. aHR:s and confidence intervals for between-person analyses on use of antipsychotics and risk of hospitalization due to psychosis relapse

|  | **Psychosis relapse hospitalization** | | | |
| --- | --- | --- | --- | --- |
| **Antipsychotic** | **aHR** | | **95% CI** | |
| Olanzapine | 0.75 | 0.66-0.86 | |  |
| Quetiapine | 0.89 | 0.71-1.11 | |  |
| Risperidone | 0.95 | 0.78-1.17 | |  |
| Aripiprazole | 0.82 | 0.68-0.99 | |  |
| AP polytherapy | 0.89 | 0.79-1.00 | |  |
| Any LAI | 0.71 | 0.59-0.86 | |  |
| Other Oral AP | 0.85 | 0.73-1.00 | |  |

|  | **Psychosis relapse hospitalization** | | | | **Any psychiatric hospitalization** | | | | **Any SUD hospitalization** | | | |
| --- | --- | --- | --- | --- | --- | --- | --- | --- | --- | --- | --- | --- |
| **Antipsychotic** | **aHR** | | **95% CI** | | **aHR** | | **95% CI** | | **aHR** | | **95% CI** | |
| Levomepromazine | 0.85 | 0.51-1.41 | | 0.96 | | 0.71-1.30 | | 1.21 | | 0.82-1.78 | |  |
| Perphenazine LAI | 0.42 | 0.26-0.69 | | 0.63 | | 0.44-0.91 | | 0.54 | | 0.32-0.92 | |  |
| Haloperidol | 1.03 | 0.74-1.44 | | 1.06 | | 0.84-1.35 | | 0.94 | | 0.69-1.29 | |  |
| Haloperidol LAI | 0.82 | 0.51-1.31 | | 0.91 | | 0.60-1.35 | | 0.99 | | 0.55-1.77 | |  |
| Zuclopenthixol LAI | 0.67 | 0.42-1.06 | | 0.70 | | 0.48-1.03 | | 1.16 | | 0.66-2.04 | |  |

Online supplement table 3. aHR:s and confidence intervals for FGA:s

| **Antipsychotic** | **Number of users** | | | **Total person years** | | **Total number of events** | |  |
| --- | --- | --- | --- | --- | --- | --- | --- | --- |
| Clozapine | 71 | | 145.63 | | 57 | |  | |
| Olanzapine | 1035 | | 883.22 | | 758 | |  | |
| Olanzapine LAI | 63 | | 51.17 | | 32 | |  | |
| Quetiapine | 358 | | 304.17 | | 266 | |  | |
| Risperidone | 286 | | 189.94 | | 179 | |  | |
| Risperidone LAI | 73 | | 66.93 | | 35 | |  | |
| Aripiprazole | 411 | | 326.66 | | 195 | |  | |
| Aripiprazole LAI | 101 | | 112.98 | | 43 | |  | |
| Paliperidone LAI | 109 | | 87.16 | | 38 | |  | |
| Ap polytherapy | 817 | | 822.63 | | 882 | |  | |
| Levomepromazine | 133 | | 59.37 | | 77 | |  | |
| Perphenazine LAI | 50 | | 40.57 | | 61 | |  | |
| Haloperidol | 186 | | 117.49 | | 147 | |  | |
| Haloperidol LAI | 49 | | 48.62 | | 44 | |  | |
| Zuclopenthixol LAI | 69 | | 56.84 | | 50 | |  | |
|  |  |  | |  | |  | |  |

Online supplement table 4.Number of users, total person years and total number of events for hospitalisation due to any psychiatric disorder

| **Antipsychotic** | **Number of users** | | | **Total person years** | | **Total number of events** | |  |
| --- | --- | --- | --- | --- | --- | --- | --- | --- |
| Clozapine | 72 | | 146.78 | | 10 | |  | |
| Olanzapine | 1037 | | 888.75 | | 496 | |  | |
| Olanzapine LAI | 63 | | 51.19 | | 14 | |  | |
| Quetiapine | 361 | | 305.05 | | 153 | |  | |
| Risperidone | 286 | | 190.04 | | 111 | |  | |
| Risperidone LAI | 73 | | 66.94 | | 15 | |  | |
| Aripiprazole | 412 | | 328.32 | | 105 | |  | |
| Aripiprazole LAI | 101 | | 112.99 | | 27 | |  | |
| Paliperidone LAI | 109 | | 87.17 | | 18 | |  | |
| Ap polytherapy | 817 | | 845.35 | | 470 | |  | |
| Levomepromazine | 135 | | 59.48 | | 50 | |  | |
| Perphenazine LAI | 52 | | 40.75 | | 40 | |  | |
| Haloperidol | 188 | | 118.22 | | 87 | |  | |
| Haloperidol LAI | 50 | | 50.6 | | 25 | |  | |
| Zuclopenthixol LAI | 72 | | 56.93 | | 32 | |  | |
|  |  |  | |  | |  | |  |

Online supplement table 5. Number of users, total person years and total number of events for hospitalisation due to substance use disorder outcome

**Online supplement table 6. Distribution of Substance use disorder hospitalizations at follow-up**

| **Substance use disorder Total N = 4667** | *Frequency* | |
| --- | --- | --- |
|  | N | % |
| Alcohol use disorder | 365 | 7.82 |
| Opioid use disorder | 168 | 3.60 |
| Cannabis use disorder | 1895 | 40.61 |
| Sedative use disorder | 78 | 1.67 |
| Cocaine use disorder | 37 | 0.79 |
| Other stimulant use disorder | 178 | 3.81 |
| Hallucinogen use disorder | 18 | 0.39 |
| Nicotine use disorder | 16 | 0.34 |
| Inhalant use disorder | 3 | 0.06 |
| Polysubstance use disorder | 1909 | 40.89 |
